# Three β-Glucuronosyltransferase Genes Involved in Arabinogalactan Biosynthesis Function in Arabidopsis Growth and Development

**DOI:** 10.3390/plants10061172

**Published:** 2021-06-09

**Authors:** Oyeyemi O. Ajayi, Michael A. Held, Allan M. Showalter

**Affiliations:** 1 Department of Environmental and Plant Biology, Ohio University, Athens, OH 45701, USA; oa715216@ohio.edu; 2 Molecular and Cellular Biology Program, Ohio University, Athens, OH 45701, USA; held@ohio.edu; 3 Department of Chemistry and Biochemistry, Ohio University, Athens, OH 45701, USA

**Keywords:** arabinogalactan, cell wall, calcium, sugar, germination, Arabidopsis, glucuronic acid

## Abstract

Arabinogalactan proteins (AGPs) contain arabinogalactan (AG) polysaccharides that are biologically relevant to plant growth processes. Here, the biochemical and physiological roles of three Golgi localized β-glucuronosyltransferase genes (*GLCAT14A*, *GLCAT14B* and *GLCAT14C*) in Arabidopsis thaliana, responsible for the addition of glucuronic acid to AG chains, were further investigated using single, double and triple *glcat14* mutant plants. These proteins were localized to the Golgi apparatus when transiently expressed in *Nicotiana benthamiana*. Sugar analysis of AGP extracts from Arabidopsis stem, leaf and siliques showed a consistent reduction in glucuronic acid in *glcat14* mutants relative to wild type, with concomitant effects resulting in tissue-specific alterations, especially in arabinose and galactose sugars. Although we observed defects in trichome branching in *glca14a/b* and *glca14a/b/c* mutants, scanning electron microscope analysis/energy dispersive microanalysis (SEM/EDX) showed no difference in the calcium content of trichomes in these mutants relative to wild type. Immunoblot analyses of the stem and leaf showed a reduction in AGPs as detected with the LM2 antibody in *glcat14a/b* and *glcat14a/b/c* mutants relative to wild type. The current work exemplifies the possibility of conducting structure-function assessment of cell wall biosynthetic genes to identify their physiological roles in plant growth and development.

## 1. Introduction

Plant cell walls are composed of structurally complex heteropolymers that are essential to plant development. Arabinogalactan-proteins (AGPs) are a family of complex proteoglycans found in the plant cell walls of all higher plants [1] and contain a large amount of arabinogalactan polysaccharide (90% *w*/*w*) attached to hydroxyproline residues in their protein cores [2]. AGPs belong to a superfamily of structural macromolecules known as the hydroxyproline-rich glycoproteins (HRGPs), of which extensins and proline-rich proteins are members with unique carbohydrate moieties central to their biological functions [3]. Out of all the HRGPs, AGPs are the most complex, and their complexity is driven by the heterogeneity of their type II arabinogalactans (AG) and the variety of their protein backbones [4]. AGPs have N-terminal signal peptides, which are cleaved to allow for secretion, and about half of AGP family members also have C-terminal glycosylphosphatidylinositol (GPI) anchors to allow for their retention and attachment to the outer leaflet of the plasma membrane [5].

AGPs are implicated in an array of plant growth and development processes, including cell expansion, somatic embryogenesis, root and stem growth, salt tolerance, hormone signaling, programmed cell death, male and female gametophyte development, and wounding/defense [4,6]. Structurally, AGs are *O*—linked to hydroxyproline residues in the AGP protein core, and each AG is characterized by the presence of a β-1,3-galactan backbone that is substituted at various *O*6 positions with β-1,6-galactan side chains [7]. These so-called type II galactan chains are further modified by arabinose (Ara), rhamnose (Rha), fucose (Fuc), xylose (Xyl) and glucuronic acid or 4–O–methyl glucuronic acid (collectively referred to as GlcA) [8,9,10]. These various AGP glycosylation reactions are catalyzed by a family of enzymes known as glycosyltransferases (GTs) [9]. While the functional roles of several AGP GTs have been identified and characterized to varying extents using molecular and biochemical approaches, the identity and biological roles of many other AGPs GTs remain to be explored.

The AGP glucuronosyltransferases or glucuronic acid transferases (GLCATs) represent one family of AGP GTs, which has been characterized to some extent. As their name implies, this family of enzymes transfer glucuronic acid (GlcA) to type II AGs and are members of the GT14 family of the carbohydrate active enzyme (CAZy). Previous work on ATGLCAT14A-C demonstrated that they transfer GlcA to both β-1,3-galactan and β-1,6-galactan side chains with distinct substrate preferences [7,11]. Recently, two additional genes/enzymes, GLCAT14D and E, were reported to be involved in the glucuronidation of type II AGs [12]. Furthermore, *glcat14a-1* and *glcat14a-2*, two allelic T-DNA insertion mutants, were reported to display the increased cell elongation phenotype in etiolated seedlings [7], a finding that was disputed in a later study [12]. In addition, multiple developmental defects such as reduced trichome branching and severe reduction in the growth of etiolated seedlings were observed in *glcat14a/b/e* mutants, including the perturbation of the calcium waves [12]. In another related study, CRISPR-Cas9-generated *glcat14a/b* and *glcat14a/b/c* mutants showed delayed seed germination, reductions in root hair length and trichome branching, reduced seed set and developmental defects in seed mucilage and pollen grains [13]. These results reinforce the critical roles that these biosynthetic enzymes play in different aspects of plant growth.

Calcium is an essential element needed for the growth and development of plants under both normal (non-stress) and stress conditions. Calcium impacts cell wall rigidity, and low calcium content weakens the cell wall to facilitate cell expansion [14]. Notably, calcium performs multiple roles in plant growth, but its effect is not limited to cell wall and membrane stability, but also serves as a secondary messenger in many aspects of plant biological processes [15]. There are speculations that AG glucuronidation of AGPs is a major source of cytosolic Ca2+, given the negatively charged GlcAs residues’ inherent ability to bind and release calcium in a pH-dependent manner at the periplasmic surface of the plasma membrane [16]. Previous work showed that some of the developmental defects in *glcat14a/b/e* mutants, specifically the trichome branching defects, were suppressed by increasing the calcium content in the growth medium [12]. This reinforces the interdependence between cell surface apoplastic calcium and GlcA residues in AGPs in regulating important signaling events critical to plant growth and development.

Here, we focus on the biochemical and physiological characterization of *glcat14* T-DNA insertion mutants of *glcat14a*, *glcat14b*, *glcat14c*, *glcat14a/c*, *glcat14b/c* along with CRISPR-Cas9 generated *glcat14a/b* and *glcat14a/b/c* mutants. Specifically, we sought to understand the degree to which the alterations in the amounts of GlcA and calcium content accounts for the mutant phenotypes identified in this study using various biochemical and molecular genetic approaches. This will bring us a step closer towards understanding the relative contributions of the *GLCAT* genes in cell wall integrity maintenance, critical to plant growth processes. 

## 2. Results

### 2.1. The CAZy GT14 Family

**Figure 1:**
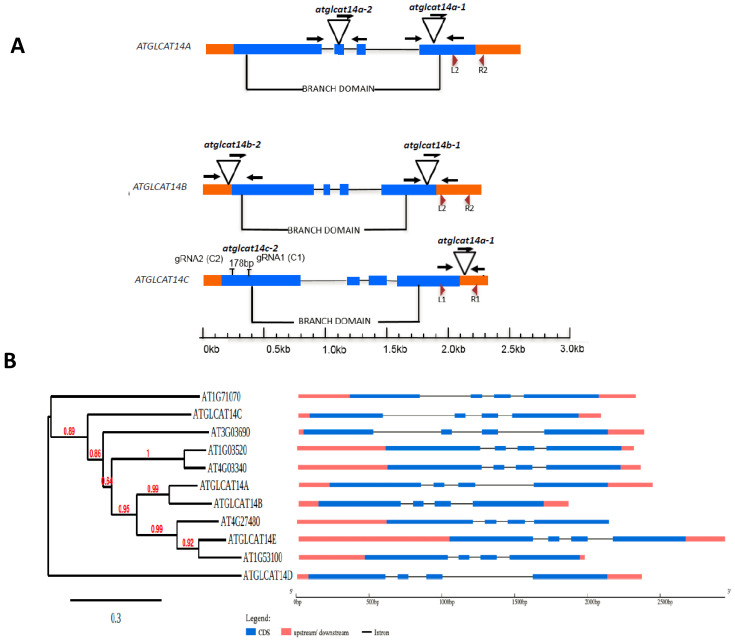

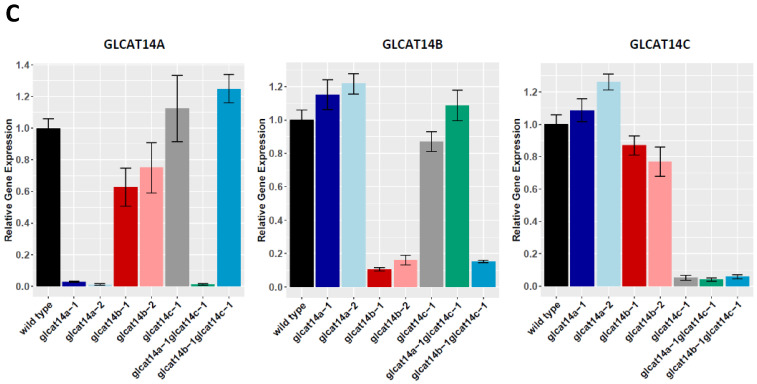
*ATGLCAT14A* (*AT5G39990*), *ATGLCAT14B* (*AT5G15050*), and *ATGLCAT14C* (*AT2G37585*) gene/genetic mutant structure, phylogenetic relationships, and expression. (**A**) Schematic drawing of the *ATGLCAT14A* (*AT5G39990*), *ATGLCAT14B* (*AT5G15050*) and *ATGLCAT14C* (*AT2G37585*) gene structure and sites of T-DNA insertions in two independent lines: *atglcat14a-1* and *atglcat14a-2* are allelic mutants for *ATGLCAT14A*; *atglcat14b-1* and *atglcat14b-2* are allelic mutants for *ATGLCAT14B*; *atglcat14c-1*, and *atglcat14c-2*, generated by CRISPR-Cas9 strategy targeting exon 1 by two guide RNAs gRNA1 (C1) and gRNA2 (C2) as indicated above [12] are allelic mutants for *ATGLCAT14C*. Exons are represented by blue boxes; introns are represented by thin gray lines and non-coding regions are represented by orange boxes. The T-DNA insertion sites for each mutant are indicated, with primer binding sites indicated by black arrows. The branch domain corresponds to PF02485 from the Pfam database is indicated above. Red arrowheads indicate primer binding sites for qRT-PCR. (**B**) Phylogenetic analysis of the GT14 gene family along with their exon-intron structure. (**C**) Relative gene expression of GLCAT14A, GLCAT14B, and GLCAT14C in Arabidopsis leaves in various genetic mutant backgrounds as determined by qRT-PCR. Transcript levels were normalized to the mean of one reference gene, the Arabidopsis Actin 2 gene, AtACT2. Averages of three biological replicates ± SE are shown.

This work sought to determine the biochemical and physiological roles of three functionally characterized GLCATs (GLCAT14A, GLCAT14B and GLCAT14C) in Arabidopsis. Previous work provided enzymatic evidence that three GLCATs (GLCAT14A, GLCAT14B, GLCAT14C) out of the 11 putative Arabidopsis GLCAT genes/enzymes in the CAZy GT14 family function in glucuronidation of AGPs [11]. More recently, GLCAT14D and GLCAT14E were reported to be involved in AG glucuronidation [12]. A common feature in verified and putative GLCATs in Arabidopsis is the presence of a highly conserved GLCAT domain [17]. Additionally, the intron-exon genetic architecture of Arabidopsis CAZy GT14 family members is conserved and includes the presence of four exons and three introns (Figure 1B). Phylogenetic analysis showed that *GLCAT14A* and *GLCAT14B* belong to the same phylogenetic clade, while *GLCAT14C*, *D* and *E* are phylogenetically distant from *GLCAT14A* and *GLCAT14B*, consistent with earlier reports [11,12]. Sequence analysis showed differences in similarities among the *GLCAT14* genes, with the lowest sequence similarity observed to be 39% (between *AT1G53100* and *ATGLCAT14D*) and highest being between *AT4G03340* and *AT1G03520*); others are nestled between these two extremes (Table S2). Interestingly, these five enzymes, ATGLCAT14A/B/C/D/E, demonstrated to be involved in the glucuronidation of type II AGs, have unique substrate preferences; ATGLCAT14A, ATGLCAT14B and ATGLCAT14C transfer GlcA onto both β- 1,3 and β- 1,6 galactan chains of shorter length, while ATGLCAT14D and ATGLCAT14E have preferences for longer galactan side chains [11,12]. 

### 2.2. glcat14 Mutant Generation and Characterization

Arabidopsis *glcat14* T-DNA insertion mutant lines were confirmed for the presence of T-DNA insertions (Figure S1A) and were subsequently used for genetic crosses to obtain higher order homozygous mutants (Figure S1B,C). Additionally, results obtained using qRT-PCR showed the absence of transcripts in these mutants (Figure 1C). We could not verify the presence of a T-DNA insertion in the SALK_051810 line belonging to *GLCAT14C*, but a CRISPR knock-out mutant (*glcat14c-2*) has a 178 bp deletion [13] in the first exon (Figure 1A) and biological phenotypes were comparable to those obtained in the *glcat14c-1* mutant (SALK_005705), as subsequently described.

### 2.3. Subcellular Localization of GLCAT14A, GLCAT14B and GLCAT14C

In the subcellular localization experiment involving ATGLCAT14A, ATGLCAT14B and ATGLCAT14C, we detected punctate vesicles that co-localized with the Golgi marker sialyltransferase short cytoplasmic tail and single transmembrane domain fused to enhanced GFP (STtmd-GFP) (Figure S3), indicating localization of ATGLCAT14A, ATGLCAT14B and ATGLCAT14C in the Golgi apparatus.

### 2.4. glcat14a/b and glcat14a/b/c Had Reduced Immunolabelling of LM2 Bound AGPs in Stem and Leaf

**Figure 2:**
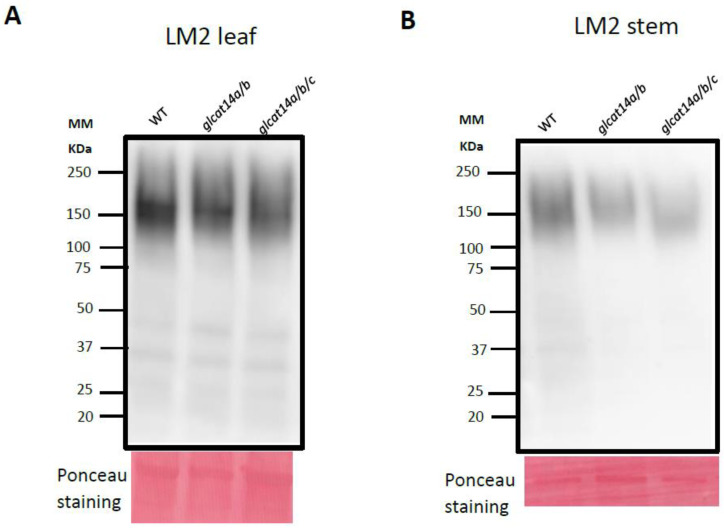
Western blot analysis of protein extracts from stem and leaf of wild type, *glcat14a/b* and *glcat14a/b/c*. Proteins (25 µg) extracted from plant materials were loaded per lane and separated by SDS-PAGE according to size, Ponceau stained, and subsequently transferred to PVDF membranes. LM2 antibody was used for immunoblotting to target β-GlcA in leaf (**A**) and stem (**B**). Molecular mass (kDa) is indicated on the left. MM, Molecular marker.

Structural modifications can affect the abundance of glycan moieties that make up the plant cell wall. These structural changes can be delineated using antibodies raised against glycan epitopes in an immunoblot analysis. The LM2 antibody, which specifically binds to β-glucuronic acid in Type II AGs in AGP protein cores, was used to immunolabel wild type, *glcat14a/b* and *glcat14a/b/c* leaf and stem protein extracts separated by SDS-PAGE prior to membrane transfer. As expected, LM2 immunolabelling generated broad smears, reflective of the heavily glycosylated nature of AGPs, and the labelling intensities of LM2-bound AGPs were greater in leaves than in stems (Figure 2A,B). In contrast to the wild type, weak signals with LM2-bound AGPs were observed in *glcat14a/b* and *glcat14a/b/c* extracts from leaves (Figure 2A) and stems (Figure 2B). Moreover, the molecular size of LM2-bound AGPs in the stem and the leaf were similar, and signal intensities were in the high molecular mass (100–250 kDa) region of the Western blot. 

### 2.5. Quantification of β-D-Gal-Yariv Precipitated AGPs in glcat14 Mutants

**Figure 3:**
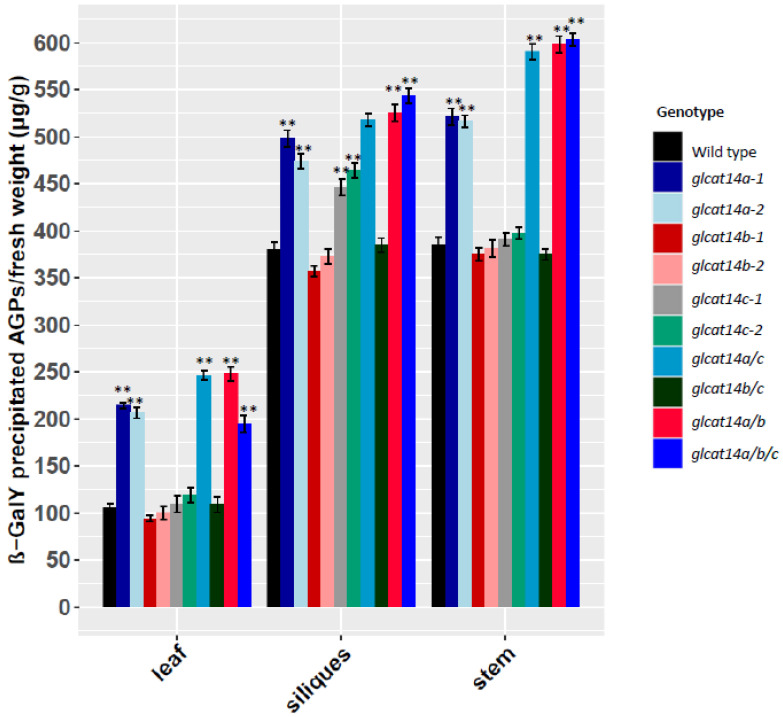
Quantification of AGPs in the various *glcat* mutants in different organs of 40-day-old Arabidopsis plants. Leaves, siliques, and stems were obtained from 40-day-old Arabidopsis plants. *glcat14a*, *glcat14a/c*, *glcat14a/b* and *glcat14a/b/c* showed significant increases in AGP content in the leaves and stems, while *glcat14a*, *glcat14c*, *glcat14a/c*, *glcat14a/b* and *glcat14a/b/c* showed increases in AGP content in siliques. AGPs were measured in micrograms per gram fresh weight. Statistical differences were determined by two-way ANOVA, followed by the Tukey’s honestly significant difference test (** *p* < 0.01); results were averages of three independent experiments.

Given the differential expression of *GLCATs* in different organs and across different developmental stages [17], we quantified the amount of Yariv precipitable AGPs from different organs of WT and *glcat14* mutants. The ability of β-D-Gal-Yariv to bind to the β-1,3-galactose backbone on AGPs was exploited to isolate and quantify AGP content as described previously [18]. Across the organs examined (rosette leaves, stems and siliques), the amounts of Yariv precipitable AGPs in the stems and siliques were greater than those observed in the rosette leaves. Specifically, in contrast to WT, we observed an increase in Yariv precipitable AGPs in *glcat14a, glcat14a/c*, *glcat14a/b* and *glcat14a/b/c* mutants in the rosette leaves and stems; however, in the siliques, the *glcat14a* mutants along with *glcat14c*, *glcat14a/c*, *glcat14a/b* and *glcat14a/b/c* mutants showed an increase in Yariv precipitable AGPs (Figure 3). The *glcat14b* and *glcat14b/c* mutants, however, did not show an increase in AGP content and were comparable to WT.

### 2.6. Monosaccharide Composition Analyses and Calcium Content

**Figure 4:**
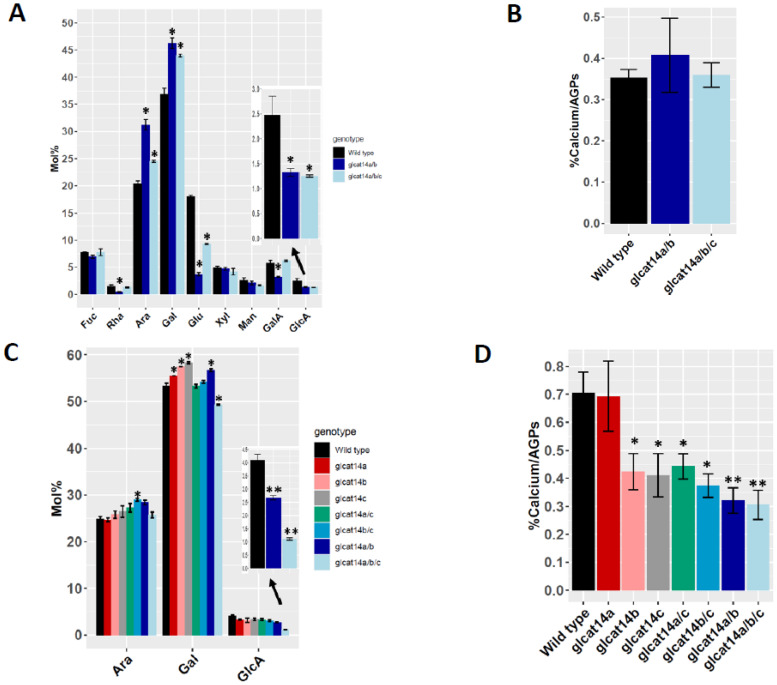

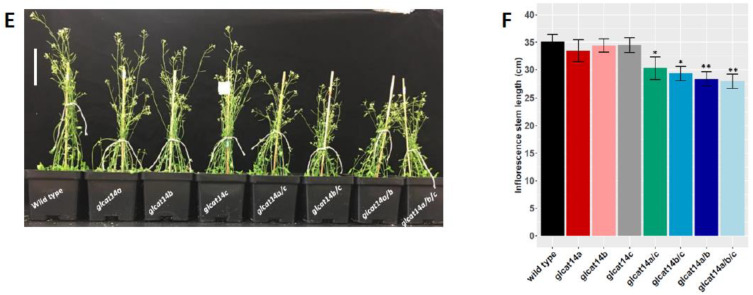
Biochemical and physiological analyses of wild type and *glcat14* mutants. (**A**) Monosaccharide composition analysis of leaf AGPs extracted from wild type and *glcat14a/b* and *glcat14a/b/c* mutants using HPAE-PAD. (**B**) Calcium content of leaf AGP extracts of wild type, *glcat14a/b*, and *glcat14a/b/c* (expressed as % calcium per AGPs). (**C**) HPAE-PAD monosaccharide composition analysis of stem AGPs extracted from wild type and *glcat14* mutants. (**D**) Calcium content of stem AGPs extracted from wild type, *glcat14a/b*, and *glcat14a/b/c* (expressed as % calcium per AGPs). For the sugar analysis, values are relative to total sugar composition (expressed as mol %) of triplicate assays ± SE. For the calcium assay, values are the mean ± standard deviation from three biological replicates. (**E**) Growth phenotypes of 40-day-old *glcat14* mutants and WT showed growth reductions in the *glcat14* double and triple mutants relative to WT; Scale bar = 12 cm. (**F**) Inflorescence stem lengths from 40-day-old wild-type and *glcat14* mutant plants. Asterisks indicate significant differences between *glcat14* mutants and WT as defined by one-way ANOVA followed by Tukey’s multiple comparison test (Student’s *t-*test, *p* < 0.05 for single asterisks, *p* < 0.01 for double asterisks), *n* = 15 per line per replicate.

AGP glycans were evaluated in stems, siliques and leaves to provide insights into organ-specific glycan composition and to understand to what extent the AGP structural modifications would impact the abundance of other AGP sugars in *glcat14* mutants relative to WT. When the plant cell wall integrity (CWI) system is compromised, adaptive changes in cellular and cell wall metabolism can alter wall composition and structure [19]. Previous work on *glcat14a* mutants reported a slight increase in galactosylation, while the GlcA content was comparable to WT in AG extracts from 14-day-old seedlings [7]. In acid-hydrolyzed AGP extracts investigated by HPAE-PAD, we observed significant changes in sugar amounts relative to WT. Specifically, in the leaves, and with the exception of *glcat14a* and *glcat14c*, we observed an increase in the amounts of galactose and minor changes in arabinose relative to the wild type (Table S3). While reductions in the amount of GlcA were observed across all genotypes in leaves, we focused on *glcat14a/b* and *glcat14a/b/c* mutants because the leaf trichome branching phenotype was only found in these mutants (discussed below) and was absent in the *glcat14* T-DNA insertion lines. In the leaf AGPs, we observed a 47% and 49% reduction in the amounts of GlcA in *glcat14a/b* and *glcat14a/b/c* mutants, respectively, relative to wild type (Figure 4A), while for stem AGPs, we observed a 35% and 73% reduction in GlcA content in *glcat14a/b* and *glcat14a/b/c* mutants, respectively, relative to wild type; the remaining genotypes had comparable GlcA amounts to WT in stem AGPs (Table S4). With the exception of *glcat14a/c* and *glcat14b/c*, which were not significantly different from WT, a reduction in galactose content in *glcat14a/b/c* in the stem was unexpected, given the significant increases in AGP content of *glcat14a/b/c* mutants in stem tissues. In addition, minor changes were observed in the amount of arabinose in *glcat14* mutants relative to WT. For the siliques, we observed a reduction in the GlcA content in *glcat14* mutants, coupled with a corresponding increase in galactose content in both single and higher order *glcat14* mutants (Table S5A). It should be noted that some residue amount of GlcA remains associated with the AGPs in all of these organs, regardless of the mutant. This suggest the involvement of other GLCATs, whose roles remain to be elucidated.

Calcium is well known as a universal signaling molecule and acts as a ‘second messenger’ in plants [20,21]. The ability of AGP to bind and release calcium in a pH-dependent manner in vitro gave rise to the AGP Ca^2+^ capacitor hypothesis [16]. The intramolecular calcium binding abilities by paired GlcA residues of AGPs was exploited to estimate the calcium content of AGPs. We investigated whether there were significant alterations in calcium bound by AGPs, possibly mediated by the reduction in the amounts of GlcA across tissues investigated using a colorimetric assay [22]. We observed that the calcium content of AGP extracts in *glcat14* mutants were similar to the WT in leaves (Figure 4B) and siliques (Table S5B); however, that was not the case for stems. While the calcium content of *glcat14a* was similar to WT, we observed a significant reduction in calcium content in *glcat14b* and *glcat14c* single mutants, and also for the higher order *glcat14* mutants in stem (Figure 4D).

### 2.7. Both Single and Double glcat14 Mutant Display Pleiotropic Growth Defects

**Figure 5:**
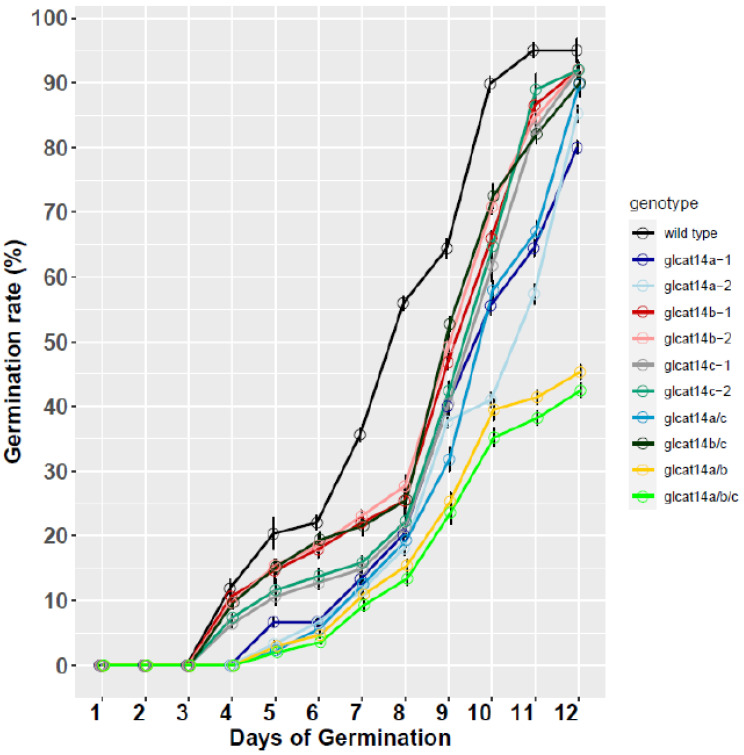
Germination percentages of the *glcat14* mutants grown in the presence of 1 µM ABA. Germination percentages of *glcat14* mutants showed delayed germination compared to wild type in normal ½ MS supplemented with 1 μM ABA, which were grown for 12 days following 3 days of stratification at 4 °C (See methods). Twenty-five seeds of each genotype were used for germination, with three replicates.

Seeds obtained from WT and *glcat14* mutant lines were grown on MS plates supplemented with or without ABA to investigate the potential roles of GLCAT14A, B, and C in seed germination, given that *GLCAT14A* and *GLCAT14B* are both highly expressed in the micropylar endosperm, and the endosperm cell wall’s weakening is repressed by ABA [13]. The *glcat14* T-DNA mutant lines germinated like WT seeds under standard growth conditions. However, when the growth media were supplemented with 1 µM ABA, we observed that the *glcat14* mutants exhibited a delay in germination (Figure 5). Similarly, seeds grown under light conditions for 9 d showed no obvious differences in root growth relative to WT (Figure S2A). Additionally, dark grown etiolated seedlings in both *glcat14* T-DNA mutant lines and the CRISPR lines showed no obvious differences in root growth relative to WT (Figure S2B). Previous work reported an increased length of hypocotyls and roots in *glcat14a* mutants of 5-day old etiolated seedlings [7]; however, it is noteworthy that our observation agrees with earlier work that showed no difference in hypocotyl length in etiolated seedlings except for *glcat14a/b/e*, which were remarkably shorter [12]. We also investigated the role of GLCAT14A, B, and C in plant height, and our results show that *glcat14a/c* and *glcat14b/c* and CRISPR lines (*glcat14a/b* and *glcat14a/b/c*) were significantly shorter than the wild type plants (Figure 4E,F).

**Figure 6:**
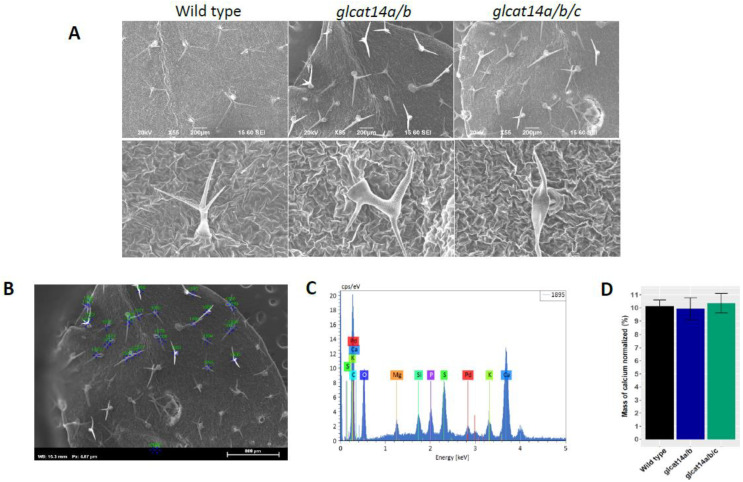
SEM and SEM/EDX elemental calcium analyses of trichomes of wild type, *glcat14a/b*, and *glcat14a/b/c* mutants. (**A**) SEM images of *glcat14a/b* and *glcat14a/b/c* mutants showing defects in trichome branching relative to WT. (**B**) Representative image of the sampling points for SEM/EDX elemental analysis of trichomes. (**C**) Representative spectral plots for the elemental composition of trichomes. (**D**) Normalized mass of calcium in WT and *glcat14a/b* and *glcat14a/b/c* mutants showed no significant difference between WT and the *glcat14* mutants.

Previous work observed that CRISPR-Cas9 triple knockouts of *GLCAT14A-C* [13] showed defects in trichome branching. A recent study similarly identified ATGLCAT14D and ATGLCAT14E as being involved in ensuring normal trichome development; this study also found that trichome defects in *glcat14* mutants were suppressed in a Ca^2+^ concentration-dependent manner [12]. Using SEM, we examined the trichome defects of *glcat14a/b* and *glcat14a/b/c* and conducted an elemental composition analysis directed at estimating the amount of calcium using the energy dispersive X-ray (EDX) microanalysis technique [23]. Energy dispersive X-ray (EDX) microanalysis is an analytical technique commonly used to give semiquantitative measurements of surface elements within the surface layer of 1–2 microns in thickness [24]. While the significant majority of the trichomes in *glcat14a/b* and *glcat14a/b/c* are one-branched instead of the two-branched trichomes characteristics of WT (Figure 6A; Table 1), EDX spectra of investigated genotypes for elemental calcium showed no significant differences in the normalized mass of calcium relative to WT (Figure 6D). It is noteworthy that none of the single and double *glcat14* T-DNA insertion mutants had defects in trichome branching, as the trichome defects were only observed in *glcat14a/b* and *glcat14a/b/c* mutants.

### 2.8. glcat14a/c Mutants Had Reduced Pollen Germination and Misshaped Pollen

**Figure 7:**
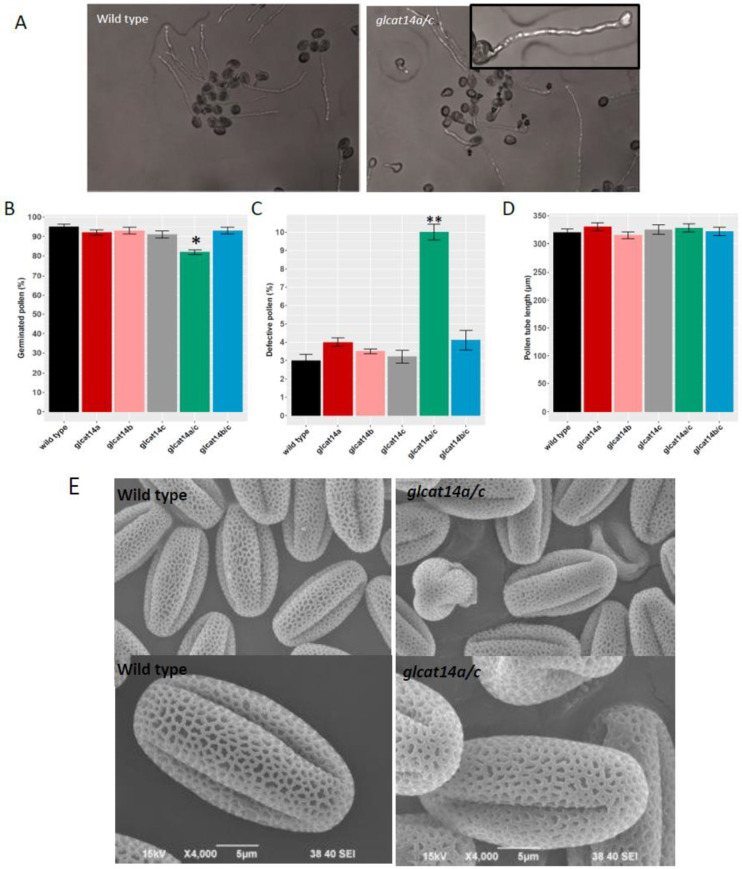
Pollen phenotypes of selected *glcat14* mutants. (**A**) In vitro pollen germination showed defective pollen (arrowheads) and pollen tube tip swelling (arrow; inset in A) in the *glcat14a/c* mutant compared to WT. (**B**) Pollen germination (%) in *glcat14* mutants and wild type showed decreased pollen germination in *glcat14a/c* mutants relative to WT. (**C**) Proportion of defective pollen was significantly higher in *glcat14a/c* mutants relative to WT. (**D**) Pollen tube lengths were similar in *glcat14* mutants and wild type. All measurements were taken 3 h after incubation of pollen grains on pollen germination media. Approximately 150 pollen grains were measured for each genotype with three replicates. (* *p* < 0.05; ** *p* < 0.01); (**E**) SEM images of *glcat14a/c* mutants showed misshaped pollen and slight alterations in the reticulate structure relative to WT.

Scanning electron micrographs and in vitro pollen germination experiments were conducted to examine the pollen morphology and pollen germination of various *glcat14* T-DNA mutants. Previous work showed that *glcat14a/b* and *glcat14a/b/c* mutants displayed significant increases in defective pollen with concomitant effects on pollen germination [13]. In this study, we observed that in addition to *glcat14a/b* and *glcat14a/b/c* mutants, pollen from the *glcat14a/c* mutant also demonstrated a significantly lower germination rate compared to WT (Figure 7B), which could be reflective of the significant increase in defective pollen in *glcat14a/c* mutants in both the in vitro pollen germination (Figure 7A,C) and SEM (Figure 7E). Although the pollen tube lengths of all *glcat14* mutants were comparable to WT (Figure 7D), an observation that is consistent with earlier studies [13], some pollen tubes of *glcat14a/c* mutants displayed tip swelling relative to WT (Figure 7A) coupled with slight alterations in the reticulate pattern of Arabidopsis pollen (Figure 7E). The role of AGPs in pollen germination was demonstrated in previous work on *agp6* and *agp11* and *agp6/11* double mutants, which displayed reduced pollen germination [25], while another report in *Torenia fournieri* demonstrated that a disaccharide sugar, β-methyl-glucuronosyl galactose (4-Me-GlcA-β-1,6-Gal), present on AGPs, makes pollen tubes competent for ovule targeting/guidance [26]. Considering that large amounts of pollen grains are produced during Arabidopsis sexual reproduction, there appears to be a significant proportion that are competent to ensure fertilization, given that the silique lengths and seed set in *glcat14a/c* mutants were comparable to WT (data not shown).

## 3. Discussion

A large number of Arabidopsis genes are predicted to contain glycosyltransferases that are involved in cell wall biosynthesis [27]. Despite these predictions, very few candidate genes have been functionally characterized. Genetic strategies deployed to elucidate glycosyltransferase functions have been hampered by functional redundancy, as many genes have been identified to play similar roles in the same and/or different organs/tissues. As a result, multiple genes have to be disrupted before biochemical or physiological roles of these cell wall genes can be deduced. Previous work identified eleven *GLCAT* genes in Arabidopsis that belong to the CAZy GT14 family [17,28]; these genes may be involved in the glucuronidation of Type II AGs, given their conserved genetic architecture and the conserved GLCAT domain present in genes [17]. While the functional roles of some *GLCAT* genes have been identified (i.e., *GLCAT14A-E*; Figure 1B), the functional roles of the remaining putative *GLCATs* remain to be verified and studied further. Work done previously using CRISPR-Cas9 knockouts of *GLCAT14A-C* showed severe pleiotropic growth defects, especially in the higher order mutants [13], while subsequent work by another group extended this work and identified developmental defects in additional Arabidopsis *glcat14* mutants that were partially suppressed by supplementing the growth medium with calcium [12]. In this report, T-DNA insertion mutants and CRISPR lines of *GLCAT14A-C* were utilized to corroborate previous findings and to obtain additional insights into the biochemical and physiological functions of *GLCAT14A-C* in Arabidopsis. Specifically, we sought to understand the degree to which the alterations in the amount of GlcA and calcium content accounts for the mutant phenotypes using biochemical and molecular genetic approaches.

Plant cell walls are important dynamic structures that need to be monitored properly to ensure their functional integrity [29]. Alterations in the polymer structure often trigger compensatory changes in the cell wall during normal growth or in changing environments. Strong support for the continuous cellular surveillance mechanism aimed at optimizing plant cell function exists [30]. The cell wall integrity maintenance mechanism constantly monitors and perceives changes in biological processes and coordinates activities to promote plant growth through the restructuring of glycan moieties [31,32]. In order to examine whether there are alterations in AGP abundance in *glcat14* mutants relative to WT, we quantified the amount of AGPs in rosette leaves, stems, and siliques using the β-Gal Yariv reagent that selectively binds to AGPs [18]. We observed that the amount of glycosylated AGPs was altered in the various organs of the *glcat14* mutants, with increased amounts of Yariv-precipitable AGP detected in siliques and stems compared to rosette leaves (Figure 3). Moreover, an increase in AGP content was detected in *glcat14a, glcat14a/c, glcat14a/b* and *glcat14a/b/c* mutants in the rosette leaves and stems, while in the siliques, *glcat14a, glcat14c, glcat14a/c, glcat14a/b* and *glcat14a/b/c* mutants showed an increase in AGP content. Although previous work reported an increase in Yariv-precipitable AGPs in CRISPR-Cas9 generated *glcat14ab* and *glcat14abc* [13], our findings indicate that the genetic knock out of GLCAT14A plays a role in the increased Yariv precipitable AGP content observed for *glcat14a/c*, *glcat14a/b* and *glcat14a/b/c* mutants. In addition, the increase in Yariv-precipitable AGP observed among *glcat14* mutants relative to the wild type might be a compensatory response that is necessary for maintaining cell wall integrity and establishing normal plant physiological functions.

Considering the observed alterations in AGP content across the investigated organs in the *glcat14* mutants, we conducted monosaccharide composition and Western blot analyses to gain additional insights into changes in the glycosylation processes in these mutants. We observed organ-specific glycosylation responses, reflected by organ-specific alterations in the galactose content in *glcat14* mutants investigated. Previous work on the sugar analysis of AGP extracts of the aerial parts of *glcat14* mutants showed an increase in the galactose content in *glcat14ab* and *glcat14abc* [13], but our findings indicate that this pattern is not the same for all organs. While we observed an increase in galactose contents in the leaves (Figure 4A) and siliques (Table S3) for *glcat14a/b* and *glcat14a/b/c*, we observed a significant reduction in galactose content in the stem for *glcat14a/b/c* mutants relative to WT (Figure 4C). Overall, the observed increase in galactose content in the *glcat14* T-DNA mutant and CRISPR mutant lines (Tables S3–S5A) centers around the idea that GlcA residues serve as a “cap” that can terminate the elongation of galactan chains and hence the absence of such GlcA residues could lead to an increase in the length of galactan chains [7]; however, this idea was challenged elsewhere [12]. Our data indicate that organ-specific cell wall changes do occur in response to structural changes, to ensure that plant cell walls can perform their biological functions in different situations [32]. 

Immunolabelling of β-GlcAs of Type II AGs showed weak immunolabelling with LM2, an antibody that targets β-GlcAs of Type II AGs, in *glcat14a/b* and *glcat14a/b/c* stem and leaf extracts compared to the wild type which had increased LM2 binding. This finding corroborates the observed reduction in GlcA in the monosaccharide composition analysis, supporting the argument that other GLCATs may be involved in the AG glucuronidation processes. The LM2 signal was detected in the high molecular mass region (Figure 2A,B), which might be a due to AGPs’ interaction with other cell wall polymers, as demonstrated in APAP1 mutants [33]. We observed weak binding of LM2 antibody in stem compared to the leaf, and this differences in LM2 binding may be due to the micro-heterogeneity of the AGP glycan in stem and leaf.

Arabidopsis *GLCAT14A-C* are highly expressed during seed germination [17]. Moreover, *GLCAT14A* and *GLCAT14B* are primarily expressed in the micropylar endosperm during seed development [34]. We observed significant delays in seed germination in the presence of 1 µm ABA in *glcat14* mutants relative to WT (Figure 5), a finding that is consistent with an earlier report [13]. ABA is reported to influence the cytosolic calcium oscillations and impact many physiological processes, such as seed germination and environmental stress responses [35,36], and such oscillations in cytoplasmic calcium concentrations could be mediated by calcium that is bound by GlcA residues in AGPs [16]. In any case, further research is needed to verify this idea.

Following the localization of ATGLCAT14A in the Golgi apparatus [7], we investigated the subcellular localizations of ATGLCAT14B and ATGLCAT14C. We observed that ATGLCAT14B-EYFP and ATGLCAT14C-EYFP signals, in addition to that of ATGLCAT14A-EYFP, were detected in punctate vesicles that co-localized with STtmd–GFP (Figure S3A–C), indicating localization of ATGLCAT14A, ATGLCAT14B and ATGLCAT14C in the Golgi apparatus. These observations are consistent with the idea that that ATGLCAT14A, ATGLCAT14B and ATGLCAT14C function in the glucuronidation of AGPs at unique positions in the type II glycan structure [12], and therefore are expected to be localized to the Golgi. 

The importance of *GLCATs* in leaf development is reflected by the significant reduction in GlcA content in *glcat14a/b* and *glcat14a/b/c* mutants relative to WT, as revealed by monosaccharide composition and Western blot analyses (Figure 2A and Figure 4A). Using SEM, we observed defects in trichome branching in *glcat14a/b* and *glcat14a/b/c* mutants (Figure 6A), and this finding is consistent with earlier work [12,13]. While most of the *glcat14a/b* and *glcat14a/b/c* trichomes were single-branched trichomes (Table 1), a much more severe trichome branching defect was reported for *glcat14a/b/e* mutants, which could be partially suppressed by supplementing the growth media with calcium [12]. Energy dispersive X-ray (EDX) analysis is an analytical technique commonly used to provide semi-quantitative measurements of surface elements within a surface layer of 1–2 microns in thickness [24]. We utilized this technique to estimate whether there were differences in the amount of calcium in trichomes of *glcat14a/b* and *glcat14a/b/c* mutants. We found that the normalized calcium masses of trichomes of *glcat14a/b* and *glcat14a/b/c* mutants were comparable to wild type based on the SEM-EDX technique (Figure 6D). Similarly, the calcium contents of leaf AGP extracts obtained from *glcat14a/b* and *glcat14a/b/c* mutants were similar to WT (Figure 4B). Despite this finding, we cannot exclude the possibility that calcium plays a role in establishing normal trichome branching and may be involved during the earlier stages of trichome development, as demonstrated earlier [12]. It is worth mentioning that most GlcA residues in leaf AGPs are methylated [10], and it remains to be determined whether certain GlcA residues added by particular GLCATs are preferentially methylated or involved in calcium binding and how such modifications impact trichome development in Arabidopsis. 

This investigation into understanding the roles of GLCAT14A, B, and C in plant growth and development identified significant reductions in plant height in *glcat14a/c*, *glcat14b/c* T-DNA insertion mutant lines relative to WT (Figure 4E,F). Previous work showed that *glcat14bc*, *glcat14ab* and *glcat14abc* were shorter than WT [13], while severe growth defects were also reported for *glcat14a/b/d* and *glcat14a/b/e* [12]. We found that *glcat14a/c* mutants were also significantly shorter than the WT but were not as severe as those observed in *glcat14a/b* and *glcat14a/b/c* mutants (Figure 4E,F). The importance of calcium was recently demonstrated by the observed hypersensitivity of the inflorescence stem lengths of some *glcat14* mutants to low calcium concentrations [12]. In addition, the significant reductions in calcium were only observed in the stem, while the calcium contents of *glcat14* mutants in leaves and siliques were comparable to WT. Considering the complex framework of plant cell wall glycans, the maintenance of cell wall structure and expansion may not be limited to the interaction of Ca^2+^ with AGP and/or pectins, as other possible interactions of calcium may exist with cell wall-modifying enzymes [20,21].

## 4. Materials and Methods

### 4.1. Plant Materials

Arabidopsis T–DNA insertion lines *atglcat14a–1* (SALK_064313) and *atglcat14a–2* (SALK_043905), *atglcat14b-1* (SALK_080923) and *atglcat14b-2* (SALK_117005), *atglcat14c-1* (SALK_005705) and *atglcat14c-2* (SALK_051810) were selected using the SIGnaL database (http://signal.salk.edu/; accessed on 26 May 2017) and were obtained from the ABRC (Arabidopsis Biological Research Centre) (http://abrc.osu.edu/; accessed on 26 May 2017) [37]. We could not verify the presence of a T-DNA insertion in SALK_051810, but a CRISPR knock-out mutant (*glcat14c-2*) in the first exon generated a 178 bp deletion [13] and was included in this study as an allelic mutant that exhibited similar phenotypes to *atglcat14c-1* (SALK_005705). Arabidopsis ecotype Col–0 was used as the wild-type for comparison. Homozygous lines were identified by PCR using primer sets identified using the T-DNA primer design tool provided by the Salk Institute (Figure 1A; Table S1) 

Homozygous *glcat14a–1* and *glcat14c-1* were crossed, and the F1 generation was selfed before screening the F2 generation to obtain *glcat14a/c.* Similarly, *glcat14b–1* and *glcat14c-1* were crossed, and the F1 generation was selfed before screening the F2 generation to obtain *glcat14b/c* (Figure S1). CRISPR-Cas9 generated *glcat14a/b* and *glcat14a/b/c* mutants (henceforth, referred simply as *glcat14a/b* and *glcat14a/b/c*) were previously generated in our lab [13] and included in this study to gain broader insight into the biological roles of the GLCATs. For the purpose of this work, *glcat14a/b* and *glcat14a/b/c*, generated by the CRISPR-Cas9 approach, are referred to as *glcat14* CRISPR lines, while the other genotypes involving T-DNA insertion mutants are referred to as *glcat14* T-DNA insertion lines.

### 4.2. Quantitative RT-PCR 

To verify whether gene transcripts exist in Arabidopsis *glcat14* T-DNA insertion lines, total RNA was extracted from Arabidopsis leaves using Trizol (Life Technologies, Grand Island, NY, USA). RNA (1 μg) was used for first-strand cDNA synthesis along with an oligo (dT20) primer and SuperScript III reverse transcriptase (Thermo Scientific). The qPCR was performed using PerfeCTa SYBR Green SuperMix (Quanta Biosciences, Gaithersburg, MD, USA) following methods described elsewhere [13]. Expression levels were calculated relative to the Arabidopsis *ACTIN2* gene. 

### 4.3. Bioinformatics

Phylogenetic analysis of CAZy GT14 family members was conducted using PhyML (http://www.phylogeny.fr/one_task.cgi?task_type=phyml, accessed on 30 May 2021), while the Gene Structure Display Server was used to determine the genetic architecture of Arabidopsis GT14 gene family (http://gsds.gao-lab.org/, accessed on 30 May 2021). 

### 4.4. AGPs Quantification Using β-D-Gal-Yariv Reagent

AGPs were extracted from rosette leaves, stems, and siliques of 40-day-old WT, *glcat14* T-DNA insertion and CRISPR lines, using the β-D-Gal-Yariv precipitation method described elsewhere [38]. The dissolved AGPs were quantified by measuring absorbance at OD_420_. Different concentrations of gum arabic (G9752, Sigma-Aldrich, St. Louis, MO, USA) dissolved in 1% CaCl_2_ were used to make the standard curve. Measurements for each genotype were done in triplicate.

### 4.5. Monosaccharide Composition Analysis by High Performance Anion Exchange Chromatography with Pulsed Amperometric Detection (HPAE-PAD)

Following the extraction of AGPs as described above, fifty microliters of 10 mg/mL AGP were hydrolyzed using 2 N TFA at 121 °C for 90 min. TFA was removed by evaporation with N_2_ gas. Samples were dissolved in 500 μL milli-Q water containing 0.2 mM cellobiose as an internal standard. A standard sugar mixture (fucose, rhamnose, arabinose, galactose, glucose, xylose, mannose, galacturonic acid, and glucuronic acid) was used for making the standard curve. Monosaccharide compositions were calculated as molar percentages (mol %). All samples and standards were subjected to high pH anion-exchange chromatography with pulsed amperometric detection (HPAE-PAD) using a Dionex PA-20 column (Thermo Fisher Scientific, Sunnyvale, CA, USA), essentially as described previously [39].

### 4.6. Calcium Binding Assay

Following the extraction of AGPs, calcium binding assays were conducted as previously described [13]. Briefly, 10 μL of 10 mg/mL AGP extracted from *glcat14* T-DNA insertion and *glcat14* CRISPR lines were used for the calcium binding assay. A commercial calcium colorimetric assay kit (MAK022, Sigma-Aldrich, St. Louis, MO, USA) was used for calcium measurement following the manufacturer’s protocol. In this assay, calcium ions from the AGP extracts form a complex with the o-cresolphthalein, which causes a color change from transparent to pink. The amount of calcium was determined using a UV spectrometer at OD_575_ and a standard curve made with different concentrations of CaCl_2_. 

### 4.7. Seed Germination 

One-month post-harvested seeds of WT and allelic *glcat14* mutant lines (*glcat14a-1*, *glcat14a-2*, *glcat14b-1, glcat14b-2*, *glcat14c-1*, *glcat14c-2*, *glcat14a/c*, *glcat14b/c, glcat14a/b*, and *glcat14a/b/c*) were used for germination experiments. Following seed sterilization, seeds were stratified at 4 °C for 3 d in the dark and were sown on ½ MS and 1% sucrose agar plates supplemented with or without 1 μM ABA. Germination percentages were counted from 1 to 12 d after sowing and approximately 25 seeds were sown for each genotype with three replicates. In another experiment to evaluate the growth of etiolated seedlings of respective genotypes, stratified seeds sown on MS plates were exposed to 4 h of fluorescent white light at 20 °C to synchronize germination before wrapping the plates in aluminum foil for growth in the darkness for 5 d at 20 °C. Root and hypocotyl length measurements at 5 d were taken and values were compared to wild type.

### 4.8. Trichome Morphology

The first two leaves of 14-day-old WT, *glcat14a/b* and *glcat14a/b/c* mutants were immersed in methanol and leaf tissues were critical point dried following methods described earlier [40] before examining using scanning electron micrography (SEM). Trichomes were examined using a JEOL JSM-6390 scanning electron microscope (Hitachi High-Technologies, Tokyo, Japan) and leaf tissues were mounted on aluminum stubs using double adhesive tapestubs and sputter coated with a palladium alloy using a Cressington 208C high-resolution sputter coater (Ted Pella Inc., Redding, CA, USA). Scanning electron microscope energy dispersive X-ray (SEM/EDX) analysis was used as a semi quantitative approach to estimate the calcium content in the trichomes of WT, *glcat14a/b* and *glcat14a/b/c* mutants following methods described previously [23].

### 4.9. In Vitro Pollen Germination

Flowers from 30-day-old WT and *glcat14a*, *glcat14b, glcat14c*, *glcat14a/c*, *glcat14b/c* plants were used for an in vitro pollen germination assay. Pollen germination medium contained 10% sucrose, 0.01% boric acid, 1 mM CaCl_2_, 1 mM Ca(NO3)_2_, 1 mM KCl, 0.03% casein enzymatic hydrolysate, 0.01% myo-inositol, 0.1 mM spermidine, 10 mM GABA, 500 μM methyl jasmonate, and 1% low-melting agarose. Pollen from each genotype was incubated on pollen germination medium. Pollen shape, pollen germination rate, and pollen tube length were measured 3 h after incubation with a Nikon phot-lab2 microscope at 10× magnification. More than 150 pollen and pollen tubes were measured in an individual experiment with three replicates.

### 4.10. Western Blotting Analysis

The total protein extraction from leaves and stems of wild type, *glcat14a/b* and *glcat14a/b/c* mutants was performed following methods described previously [41]. Total protein supernatants from the samples extracted were quantified by the Bradford method [42]. Twenty-five micrograms (25 μg) of crude protein extract of wild type, *glcat14a/b* and *glcat14a/b/c* mutants were separated using 10% mini-PROTEAN^®^ TGX™ (456-1033; Bio-Rad, Hercules, CA, USA, https://www.bio-rad.com, accessed on 30 May 2021), gel electrophoresis and transferred onto a 0.45-μm Immun-Blot low florescence PVDF membrane (1620260; Bio-Rad) using the wet transfer system at a constant current of 100V for 45 min. The blots were blocked for 1 h (3% BSA, in 1× Tris-buffered saline with Tween (150 mM NaCl, 20 mM Tris base, 0.1% Tween 20; TBST)), probed with 1:10 LM2 monoclonal rat IgM primary antibody (Carbosource, https://www.ccrc.uga.edu, accessed on 30 May 2021) in blocking buffer for 1 h, washed three times with 1× TBST for 15 min, probed with goat anti-rat IgG H + L secondary antibody (PI31629; FisherScientific), washed thrice with 1× TBST for 10 min, once with 1× TBS for 10 min, and treated with chemiluminescent substrate (Clarity ^TM^ ECL substrate, 1705060S; Biorad). A ChemiDoc imaging system (Bio-Rad) was used for image acquisition. PVDF membranes were incubated for 10 min with Ponceau S solution (0.1% (*w*/*v*) Ponceau S; 5% (*v*/*v*) acetic acid) to stain for total proteins.

### 4.11. Cloning Procedures and Subcellular Localizations of GLCAT14A, GLCAT14B and GLCAT14C

Full-length sequenced *GLCAT14A*, *GLCAT14B* and *GLCAT14C* CDS, flanked by the sites *KpnI* were subcloned into pSAT6 vector to generate pSAT6-*GLCAT14A*-EYFP and pSAT6-*GLCAT14C*-EYFP constructs containing *PI-PSPI* restriction sites. Following the digestion of the new constructs (pSAT6-*GLCAT14A*-EYFP, pSAT6-*GLCAT14B*-EYFP and pSAT6-*GLCAT14C*-EYFP) with *PI-PSPI*, the fragment was introduced into the pPZP-RCS-nptII binary vector [43]. The binary vector containing the constructs was transformed into *Agrobacterium* strain GV3101 before infiltration using 5-week old tobacco leaves grown at 22–24 °C [44]. Golgi co-localization was confirmed by using the Golgi marker sialyltransferase short cytoplasmic tail and single transmembrane domain fused to enhanced GFP (STtmd-GFP). Transformed plants were incubated under normal growth conditions and imaged 3 days post-infiltration using an upright Zeiss LSM 510 META laser scanning confocal microscope (Jena, Germany), with a 40× oil immersion lens and an argon laser. Both GFP and E-YFP were imaged using 458 and 488 nm excitation wavelengths, respectively.

## 5. Conclusions

This study corroborates and extends our understanding of the molecular dynamics of GlcA and calcium in AGPs and how they influence plant physiological processes. Although we observed a significant reduction in glucuronidation that differs across organs in *glcat14* mutants, it remains to be determined which of the GlcA residues added by the various GLCATs are modified by calcium or methyl groups or are cross-linked with other structurally complex heteropolymers such as pectins [33] to perform their cellular function. An understanding of which GlcA residues are modified and/or unmodified and how such modifications (or lack of modifications or crosslinking with other cell wall polymers) impact plant physiological functions remains to be established. Future research aiming to address such questions could potentially increase our understanding of AGP-Ca^2+^ function and its possible multifunctional role in plant growth processes.

## Figures and Tables

**Table 1:** Percentage of trichome branches in wild type, *glcat14a/b* and *glcat14a/b/c*.

Number of Trichome Branches				
Genotype	1	2	3	Total
**Wild Type**	3.19	92.6	4.26	94
***glcat14a/b***	72.06	26.47	1.47	68
***glcat14a/b/c***	90.2	7.84	1.96	51

## Data Availability

Not applicable.

## Supplementary Materials

The following are available online at https://www.mdpi.com/article/10.3390/plants10061172/s1, Figure S1: PCR screening of *glcat14* T-DNA insertion mutants. Figure S2: Growth measurements of *glcat14* mutants and wild type. Figure S3: Subcellular localization of AtGLCAT14A, AtGLCAT14B and AtGLCAT14C. Table S1: List of primers used for mutant characterization using PCR. Table S2: Amino acid sequence similarity matrix among GT14 family. Table S3: Monosaccharide composition analysis of Yariv-precipitated AGPs extracted from rosette leaves of 40-day-old *glcat14* mutants and WT (Col-0). Table S4: Monosaccharide composition analysis of AGPs extracted from stems of 40-day-old *glcat14* mutants and WT (Col-0). Table S5: Monosaccharide composition analysis of AGPs extracted from siliques of 40-day-old *glcat14* mutants and WT (Col-0).
