# Supplementary material for: Three β-Glucuronosyltransferase Genes Involved in Arabinogalactan Biosynthesis Function in Arabidopsis Growth and Development
Source: Plants (Basel). 2021 Jun 9;10(6):1172. doi: 10.3390/plants10061172 (PMC8227792; doi:10.3390/plants10061172)
Supplement: Supplementary file 1 [file plants-10-01172-s001.zip › plants-1252806-supplementary.pdf]

**Three β-Glucuronosyltransferase Genes Involved in Arabinogalactan Biosynthesis Function in Arabidopsis Growth and Development**

Oyeyemi O. Ajayi[1,2,] Michael A. Held[2,3] and Allan M. Showalter[1,2]
1. Department of Environmental and Plant Biology, Ohio University, Athens, OH 45701, USA
2. Molecular and Cellular Biology Program, Ohio University, Athens, OH 45701, USA
3. Department of Chemistry and Biochemistry, Ohio University, Athens, OH 45701, USA
*__Corresponding author__: Allan M. Showalter, Email: showalte@ohio.edu

**Supplemental Figures and Tables**

**A**

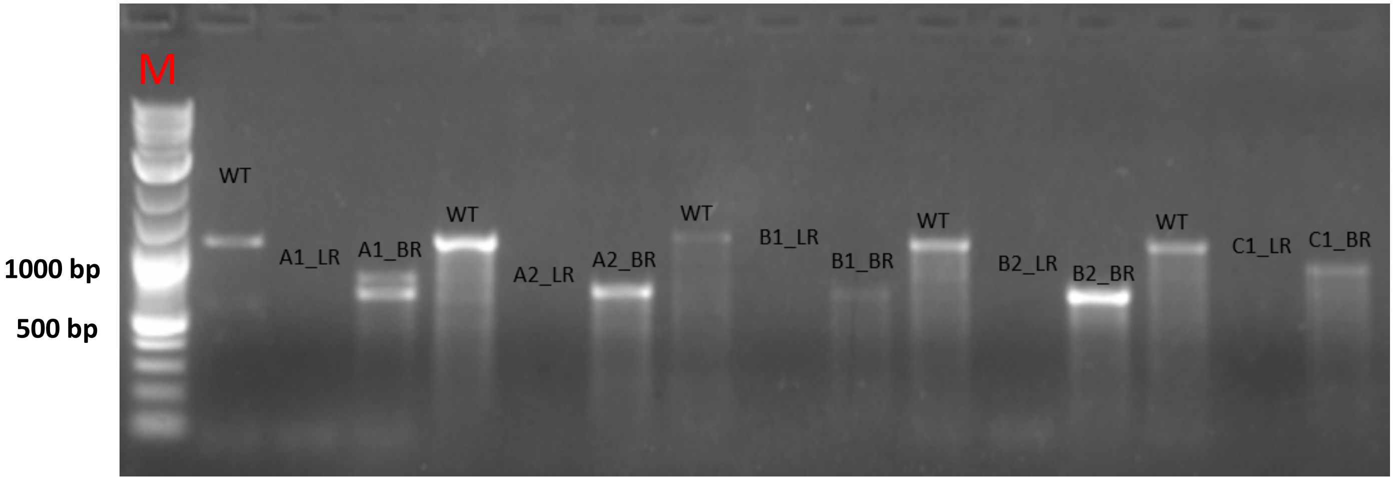
M
1000 bp
500 bp
WT
A1_LR
A1_BR
WT
A2_LR
A2_BR
WT
B1_LR
B1_BR
WT
B2_LR
B2_BR
WT
C1_LR
C1_BR

**B**

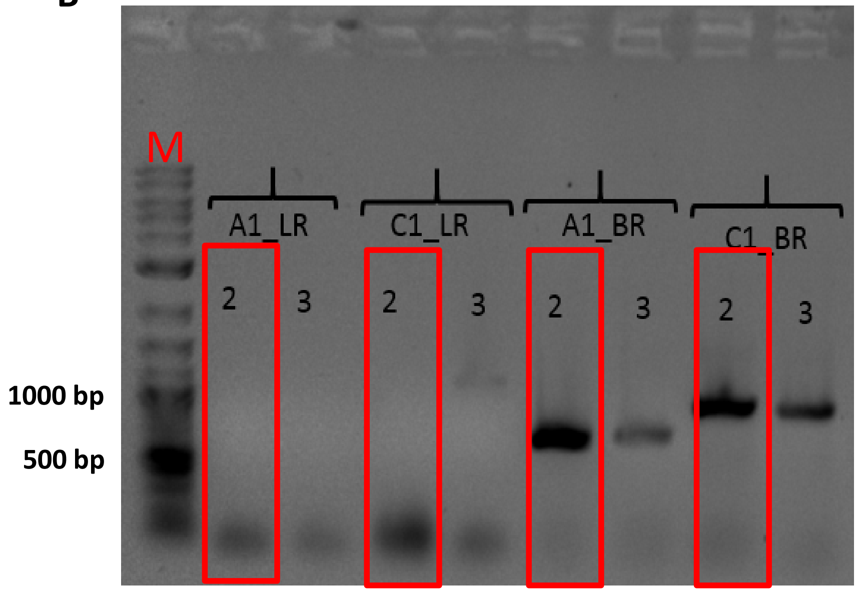
M
1000 bp
500 bp
A1_LR
C1_LR
A1_BR
C1_BR
2
3
2
3
2
3
2
3

**C**

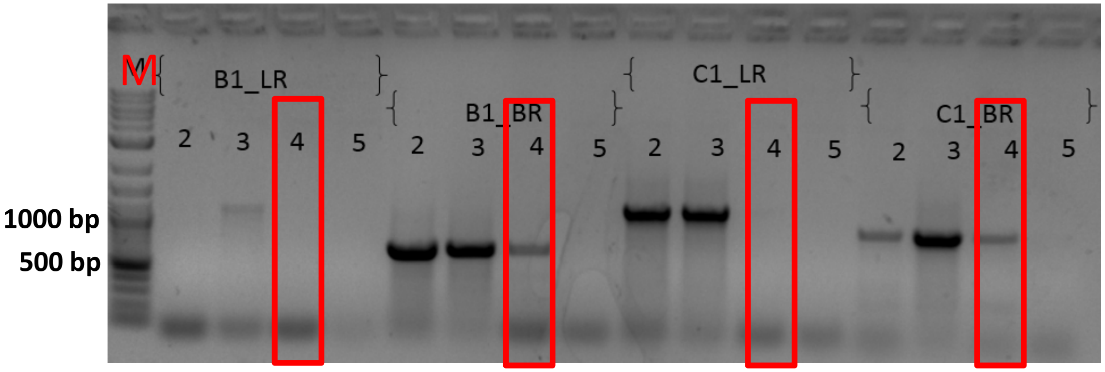
M
1000 bp
500 bp
B1_LR
B1_BR
C1_LR
C1_BR
2
3
4
5
2
3
4
5
2
3
4
5
2
3
4
5

**Figure S1: PCR screening of single mutants (A) and *glcat14a-1glcat14c-1* double mutant (B) and *glcat14b-1glcat14c-1* double mutant (C).** A1, A2, B1, B2, C1 corresponded to *glcat14a-1*, *glcat14a-2*, *glcat14b-1*, *glcat14b-2*, *glcat14c-1* single mutants respectively; LR – Left and Right primers were used for PCR screening, BR – border primer and right primer were used for PCR screening. Red boxes in panel B and C represents the confirmed homozygous double mutants, lane 2 in panel B was confirmed homozygous *glcat14a-glcat14c-1* (*glcat14a/c*) mutants while lane 4 in panel C was confirmed homozygous *glcat14b-1glcat14c-1* (*glcat14b/c*) mutants; M- Molecular ladder.

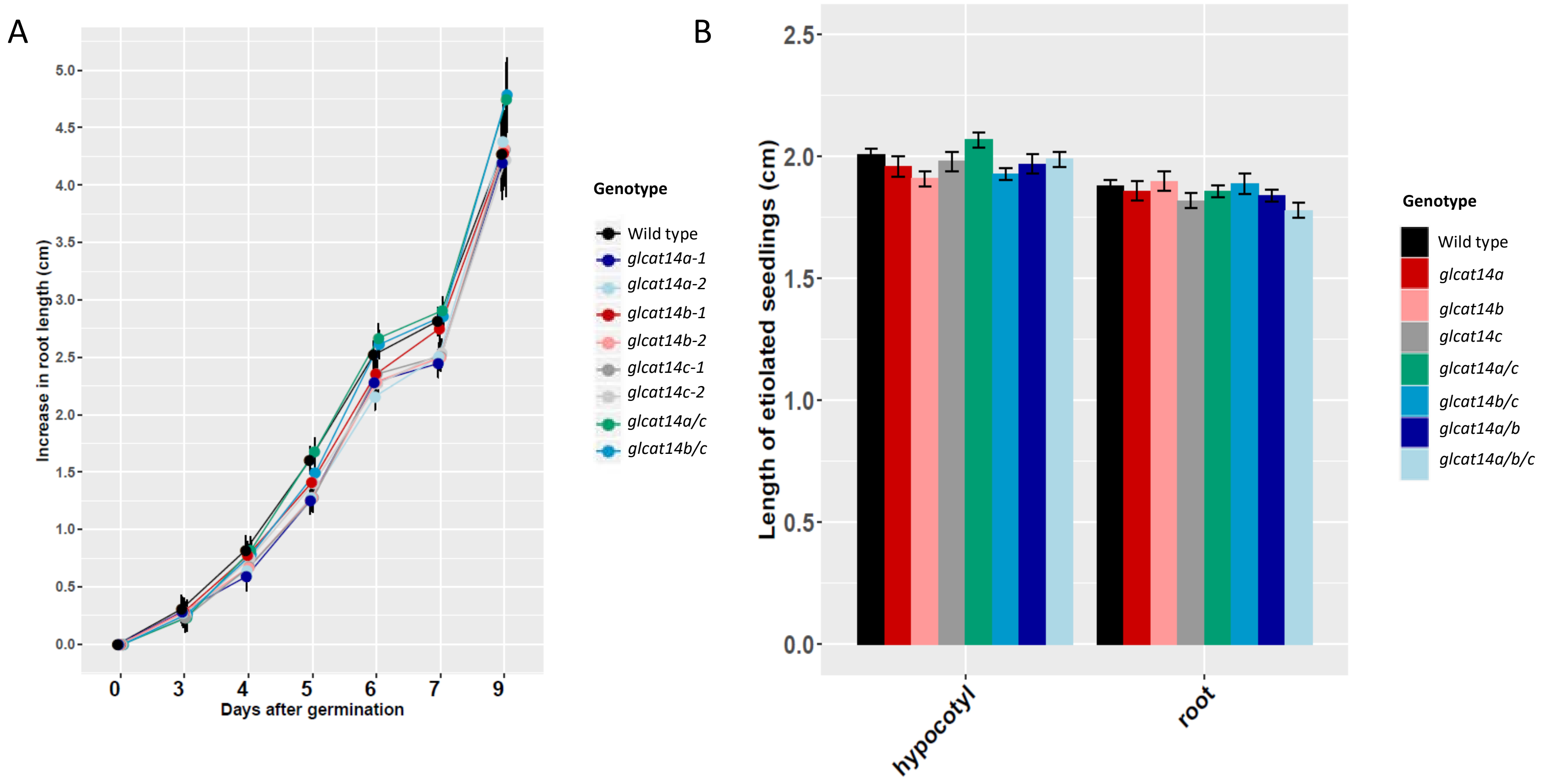


**Figure S2: Growth measurements of *glcat14* mutants and wild type.** A, Root length phenotypes of wild type and *glcat14* mutants. Results showed that the *glcat14* mutants were comparable to wild type. B, Length of roots and hypocotyls from seedlings grown in the dark for 5 days was compared to the wild type (n > 40). No significant differences were observed between wild type and *glcat14* mutants.

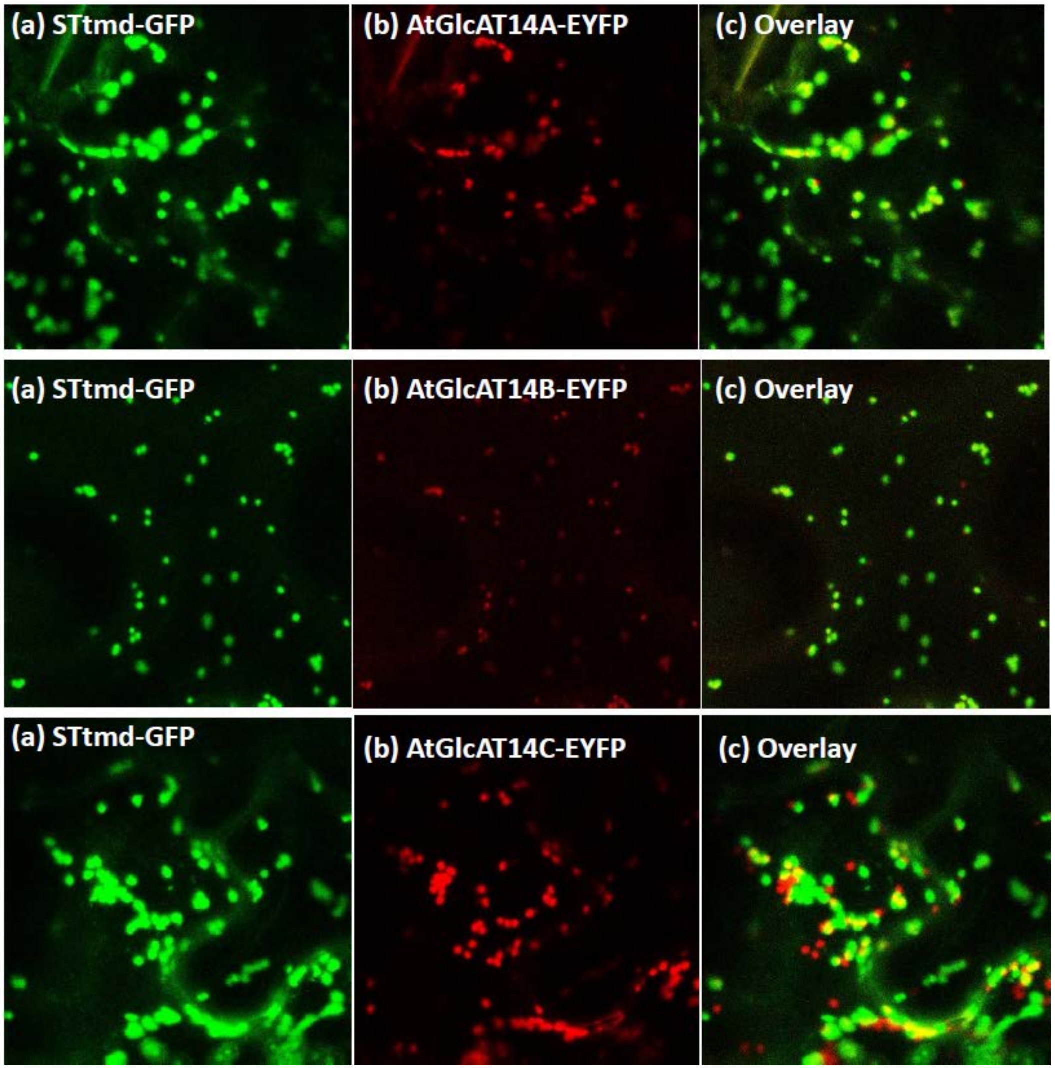


**Figure S3: Subcellular localization of *AtGlcAT14A, AtGlcAT14B* and *AtGlcAT14C*.** Subcellular localizations of *AtGlcAT14A-EYFP, AtGlcAT14B-EYFP* and *AtGlcAT14C-EYFP*. STtmd-GFP represents sialyltransferase short cytoplasmic tail and single transmembrane domain fused to enhanced GFP, while *AtGlcAT14A, AtGlcAT14B* and *AtGlcAT14C* were fused to enhanced YFP to generate fusion

protein, namely *AtGlcAT14A-EYFP, AtGlcAT14B-EYFP* and *AtGlcAT14C-EYFP.* Overlay in c, represents the overlaid image of panels a and panel b.

| Table S1: List of primers used for mutant characterization using PCR. | | |
|---|---|---|
| Purpose | | |
| PCR | Sequence | |
| glcat14a-1_LP | TAGCCACACGACATGTTCAAG | |
| glcat14a-1_RP | ACTATGCTGCAGAATTTTTAAGTTC | |
| glcat14a-2_LP | ATTGGTTCAATCTTCGCTTTG | |
| glcat14a-2_.RP | TCAACCAATGAGAAATGGAGC | |
| glcat14b-1_LP | TGATGTTTTCTGGGTTTCACAG | |
| glcat14b-1_RP | AGTGACCATCAATAGGCCCTC | |
| glcat14b-2_LP | GCAGCGTGAAGTGTATTAGCC | |
| glcat14b-2_RP | TTGCGTGTTTAAGGGATTAGC | |
| glcat14c-1_LP | ACGACGTAATCGAATTGGATG | |
| glcat14c-1_RP | TGTAACGGTTGAGAATTTCCG | |
| LBb1.3 | ATTTTGCCGATTTCGGAAC | |
| qPCR primers | | |
| At5g39990.L2 | GGATGATAACACCTGGCGGT | |
| At5g39990.R2 | AAGGCATGTTGTGTGGAGCA | |
| At5g15050.L2 | TCCGGGTGGTTGGTGTATTG | |
| At5g15050.R2 | GCCAAACTTTAGGTCGGCAA | |
| At2g37585_L2 | AGGCTTGAGAAGCTCATGGTT | |
| At2G37585_R2 | AGAACTCTTTAGACGAATGAGACCA | |

Table S2: Amino acid sequence similarity matrix among GT14 family.

| | ATGLCAT14A | ATGLCAT14B | ATGLCAT14C | ATGLCAT14D | ATGLCAT14E | AT1G53100 | AT1G03520 | AT1G71070 | AT3G03690 | AT4G03340 | AT4G27480 |
|---|---|---|---|---|---|---|---|---|---|---|---|
| ATGLCAT14A | | 72.12 | 54.19 | 49.16 | 58.24 | 53.58 | 53.12 | 49.43 | 57.59 | 55.11 | 59.29 |
| ATGLCAT14B | | | 54.15 | 49 | 55.61 | 59.6 | 55.5 | 49.14 | 58.17 | 54.89 | 57.86 |
| ATGLCAT14C | | | | 42.82 | 53.24 | 49.33 | 53.6 | 46.42 | 54.75 | 52.97 | 50.29 |
| ATGLCAT14D | | | | | 39.76 | 38.85 | 45.45 | 45 | 44.74 | 45.11 | 41.53 |
| ATGLCAT14E | | | | | | 73.93 | 52.05 | 47.86 | 63.82 | 50.24 | 68.47 |
| AT1G53100 | | | | | | | 56.45 | 44.7 | 58.77 | 50.6 | 64.82 |
| AT1G03520 | | | | | | | | 47.28 | 53.71 | 76.06 | 52.47 |
| AT1G71070 | | | | | | | | | 47.94 | 43.77 | 42.52 |
| AT3G03690 | | | | | | | | | | 53.89 | 54.36 |
| AT4G03340 | | | | | | | | | | | 52.26 |

Table S3: Monosaccharide composition analysis of AGPs extracted from leaf tissue of 40-day-old *glcat14* mutants and WT (Col-0). Values are relative to total sugar composition (expressed as mol %) of triplicate assays ± SE.

| | **Fuc** | **Rha** | **Ara** | **Gal** | **Glu** | **Xyl** | **Man** | **GalA** | **GlcA** |
|---|---|---|---|---|---|---|---|---|---|
| wild type | 1.15 ± 0.39 | 9.35 ± 0.59 | 26.56 ± 0.57 | 36.60 ± 0.78 | 4.36 ± 0.57 | 2.67 ± 0.41 | 3.0 ± 0.8 | 11.20 ± 0.97 | 5.05 ± 0.42 |
| *glcat14a* | 1.05 ± 0.29 | 5.82 ± 0.16 | 27.84 ± 0.78 | 37.19 ± 0.21 | 5.18 ± 0.41 | 2.59 ± 0.15 | 3.87 ± 0.14 | 12.94 ± 0.89 | 3.48 ± 0.72 |
| *glcat14b* | 1.28 ± 0.21 | 9.16 ± 0.92 | 23.03 ± 0.53 | 40.59 ± 0.34 | 5.38 ± 0.57 | 3.99 ± 0.56 | 3.48 ± 0.15 | 10.28 ± 0.92 | 2.77 ± 0.35 |
| *glcat14c* | 0.93 ± 0.19 | 7.88 ± 0.63 | 23.44 ± 0.70 | 37.69 ± 0.68 | 8.65 ± 0.17 | 4.98 ± 0.26 | 4.17 ± 0.61 | 9.25 ± 0.16 | 2.97 ± 0.58 |
| *glcat14a/c* | 1.09 ± 0.72 | 5.51 ± 0.35 | 23.15 ± 0.12 | 46.35 ± 0.34 | 9.93 ± 0.48 | 2.04 ± 0.45 | 3.19 ± 0.38 | 5.85 ± 0.35 | 2.85 ± 0.27 |
| *glcat14b/c* | 2.45 ± 0.54 | 8.44 ± 0.69 | 24.85 ± 0.87 | 46.95 ± 0.71 | 3.77 ± 0.23 | 2.23 ± 0.14 | 3.13 ± 0.75 | 5.66 ± 0.81 | 2.47 ± 0.52 |
| *glcat14a/b* | 1.25 ± 0.39 | 3.87 ± 0.23 | 28.84 ± 0.45 | 46.69 ± 0.88 | 5.24 ± 0.32 | 1.85 ± 0.47 | 2.85 ± 0.88 | 8.23 ± 0.56 | 1.14 ± 0.12 |
| *glcat14a/b/c* | 1.67 ± 0.35 | 4.25 ± 0.29 | 26.34 ± 0.41 | 52.44 ± 0.47 | 4.34 ± 0.65 | 0.93 ± 0.2 | 1.96 ± 0.45 | 5.89 ± 0.44 | 2.15 ± 0.25 |

Table S4: Monosaccharide composition analysis of AGPs extracted from stem tissue of 40-day-old *glcat14* mutants and WT (Col-0). Values are relative to total sugar composition (expressed as mol %) of triplicate assays ± SE.

| | **Fuc** | **Rha** | **Ara** | **Gal** | **Glu** | **Xyl** | **Man** | **GalA** | **GlcA** |
|---|---|---|---|---|---|---|---|---|---|
| wild type | 0.97 ± 0.27 | 2.16 ± 0.22 | 24.92 ± 0.59 | 53.33 ± 0.72 | 6.80 ± 0.34 | 1.28 ± 0.31 | 1.34 ± 0.4 | 5.05 ± 0.47 | 4.09 ± 0.49 |
| *glcat14a* | 0.87 ± 0.14 | 1.67 ± 0.17 | 24.65± 0.71 | 55.51 ± 0.41 | 4.26 ± 0.80 | 0.93 ± 0.24 | 1.06 ± 0.25 | 7.72 ± 0.48 | 3.28 ± 0.23 |
| *glcat14b* | 1.29 ± 0.24 | 1.51 ± 0.34 | 25.78 ± 0.34 | 57.48 ± 0.70 | 1.79 ± 0.58 | 0.86 ± 0.58 | 1.19 ± 0.26 | 6.89 ± 0.16 | 3.16 ± 0.26 |
| *glcat14c* | 0.92 ± 0.22 | 1.93 ± 0.14 | 26.47 ± 0.40 | 58.32 ± 0.71 | 2.22 ± 0.18 | 0.63 ± 0.25 | 1.15 ± 0.19 | 4.94 ± 0.28 | 3.37 ± 0.86 |
| *glcat14a/c* | 0.88 ± 0.43 | 1.72 ± 0.31 | 27.27± 0.36 | 53.30 ± 0.39 | 5.29 ± 0.14 | 3.21 ± 0.18 | 1.15 ± 0.17 | 3.83 ± 0.21 | 3.31 ± 0.28 |
| *glcat14b/c* | 0.91 ± 0.15 | 2.03 ± 0.53 | 28.99 ± 0.67 | 54.17 ± 0.75 | 3.76 ± 0.58 | 0.59 ± 0.17 | 1.30 ± 0.22 | 5.10 ± 0.26 | 3.12 ± 0.47 |
| *glcat14a/b* | 1.22 ± 0.28 | 1.05 ± 0.23 | 28.40 ± 0.48 | 56.72 ± 0.93 | 2.62 ± 0.13 | 1.11 ± 0.20 | 1.41 ± 0.23 | 4.77 ± 0.19 | 2.67 ± 0.22 |
| *glcat14a/b/c* | 1.33 ± 0.12 | 1.11 ± 0.25 | 25.74 ± 0.86 | 49.30 ± 0.88 | 11.66 ± 0.55 | 0.89 ± 0.17 | 0.68 ± 0.27 | 8.12 ± 0.37 | 1.11 ± 0.26 |

Table S5: (A) Monosaccharide composition analysis of AGPs extracted from siliques tissue of 40-day-old *glcat14* mutants and WT (Col-0). Values are relative to total sugar composition (expressed as mol %) of triplicate assays ± SE; (B) Calcium analysis of AGPs extracted from silique tissue of 40-day-old *glcat14* mutants and WT (Col-0) expressed as percentage of calcium per AGPs, SE= Standard error.

(A)

| | **Fuc** | **Rha** | **Ara** | **Gal** | **Glu** | **Xyl** | **Man** | **GalA** | **GlcA** |
|---|---|---|---|---|---|---|---|---|---|
| wild type | 1.15 ± 0.39 | 9.35 ± 0.59 | 26.56 ± 0.57 | 36.60 ± 0.78 | 4.36 ± 0.57 | 2.67 ± 0.41 | 3.0 ± 0.8 | 11.20 ± 0.97 | 5.05 ± 0.42 |
| *glcat14a* | 1.05 ± 0.29 | 5.82 ± 0.16 | 27.84 ± 0.78 | 37.19 ± 0.21 | 5.18 ± 0.41 | 2.59 ± 0.15 | 3.87 ± 0.14 | 12.94 ± 0.89 | 3.48 ± 0.72 |
| *glcat14b* | 1.28 ± 0.21 | 9.16 ± 0.92 | 23.03 ± 0.53 | 40.59 ± 0.34 | 5.38 ± 0.57 | 3.99 ± 0.56 | 3.48 ± 0.15 | 10.28 ± 0.92 | 2.77 ± 0.35 |
| *glcat14c* | 0.93 ± 0.19 | 7.88 ± 0.63 | 23.44 ± 0.70 | 37.69 ± 0.68 | 8.65 ± 0.17 | 4.98 ± 0.26 | 4.17 ± 0.61 | 9.25 ± 0.16 | 2.97 ± 0.58 |
| *glcat14a/c* | 1.09 ± 0.72 | 5.51 ± 0.35 | 23.15 ± 0.12 | 46.35 ± 0.34 | 9.93 ± 0.48 | 2.04 ± 0.45 | 3.19 ± 0.38 | 5.85 ± 0.35 | 2.85 ± 0.27 |
| *glcat14b/c* | 2.45 ± 0.54 | 8.44 ± 0.69 | 24.85 ± 0.87 | 46.95 ± 0.71 | 3.77 ± 0.23 | 2.23 ± 0.14 | 3.13 ± 0.75 | 5.66 ± 0.81 | 2.47 ± 0.52 |
| *glcat14a/b* | 1.25 ± 0.39 | 3.87 ± 0.23 | 28.84 ± 0.45 | 46.69 ± 0.88 | 5.24 ± 0.32 | 1.85 ± 0.47 | 2.85 ± 0.88 | 8.23 ± 0.56 | 1.14 ± 0.12 |
| *glcat14a/b/c* | 1.67 ± 0.35 | 4.25 ± 0.29 | 26.34 ± 0.41 | 52.44 ± 0.47 | 4.34 ± 0.65 | 0.93 ± 0.2 | 1.96 ± 0.45 | 5.89 ± 0.44 | 2.15 ± 0.25 |

(B)

| | **(Ca/AGP) % ± SE** |
|---|---|
| wild type | 0.63 ± 0.14 |
| *glcat14a* | 0.64 ± 0.11 |
| *glcat14b* | 0.64 ± 0.19 |
| *glcat14c* | 0.65 ± 0.15 |
| *glcat14a/c* | 0.67 ± 0.29 |
| *glcat14b/c* | 0.67 ± 0.13 |
| *glcat14a/b* | 0.71 ± 0.31 |
| *glcat14a/b/c* | 0.77 ± 0.29 |
